# Incidence and outcome of immune checkpoint-induced pneumonitis in oncology patients with history of pulmonary disease

**DOI:** 10.3389/fonc.2023.1283360

**Published:** 2023-10-24

**Authors:** Emily Allen, Godsfavour Umoru, Veronica Ajewole, Eric Bernicker

**Affiliations:** ^1^ Hematology/Oncology Department, Houston Methodist Hospital Texas Medical Center, Houston, TX, United States; ^2^ Texas State University College of Pharmacy and Health Sciences, Houston, TX, United States

**Keywords:** immune checkpoint-induced pneumonitis, immunotherapy, pulmonary disease, immune checkpoint inhibitors, PD-1/PD-L1 inhibitors

## Abstract

**Background:**

Immune checkpoint-induced pneumonitis (ICIP) is one of the most fatal adverse events caused by immune checkpoint inhibitors (ICI) and accounts for 35% of anti-PD-[L]1-related deaths. Risk factors including thoracic radiation and use of EGFR tyrosine kinase inhibitors have been identified as contributors to ICIP development. However, there has been very limited information on obstructive pulmonary disease as a risk factor.

**Objective:**

The purpose of this study is to evaluate the incidence and management of ICIP in a cohort of patients with pre-existing obstructive pulmonary disease.

**Methods:**

This retrospective, descriptive study, includes data from 139 patients between January 1, 2017 and August 31, 2022. Patients included were adult patients 18 years or older, received at least 2 cycles of an immune checkpoint inhibitor, and had a history of an obstructive pulmonary disorder prior to administration. Patients were excluded if they had literature-established risk factors for pneumonitis.

**Results:**

The incidence of ICIP was 7.19% (10 out of 139 patients). From a management perspective, 90% of patients had immunotherapy held, 40% received oral steroids, and 70% received intravenous steroids at the time of ICIP identification. After receiving treatment for the initial episode of ICIP, 6 patients restarted immunotherapy and 3 (50%) subsequently experienced a recurrent episode. One patient experienced grade 4 ICIP event and subsequently died from respiratory failure attributed to ICIP.

**Conclusion:**

These findings indicate that a pre-existing history of an obstructive pulmonary disorder may be a risk factor for the development of ICIP and subsequent recurrence of ICIP when rechallenged.

## Introduction

The use of immune checkpoint inhibitors (ICI) such as pembrolizumab, nivolumab, ipilimumab, durvalumab, atezolizumab, and cemiplimab has been increasing over the past several years. These agents continue to receive FDA approval across a variety of cancer types and function by downregulating inhibitory pathways on T cells, leading to increased immune system activation and T-cell recognition and attack of tumor cells. Since the introduction of these agents, pneumonitis has proven to be one of the most common fatal adverse effects seen and account for 35% of anti-PD-[L]1-related deaths (1, 2). The incidence in literature has been reported to be 2.5-5% with monotherapy immune checkpoint inhibitor use (mean onset of 2.8 months) but recent data have suggested that the overall incidence and time to onset may be higher outside of clinical trial settings (3–6). However, there is paucity of data available on how to stratify patients at significant risk for development of ICI-pneumonitis (ICIP) and whether specific tumor characteristics, histology, or combination treatments increase the incidence. A subgroup analysis of KEYNOTE-001 study which investigated utilization of pembrolizumab for the treatment of metastatic non-small cell lung cancer (NSCLC) found that pneumonitis occurred more frequently in patients with a history of asthma and chronic obstructive pulmonary disease (COPD) than in those without this history (5.4 vs. 3.1%) (4). However, given the underrepresentation of patients with underlying lung diseases in clinical trials, it is unknown if certain pre-existing obstructive lung diseases impact the risk for developing ICIP.

Furthermore, from a management standpoint, the optimal dose, duration, and type of immunosuppressive treatment for steroid refractory ICIP have not been clearly elucidated in literature. For instance, in patients who achieve clinical resolution from ICIP, there is limited data on pre-disposing factors for recurrence of ICIP upon rechallenge with immune checkpoint inhibitors. Moreover, we do not have consensus across various guidelines on the efficacy and timing of steroid-sparing agents (e.g. infliximab, cyclophosphamide, IVIG) in patients who develop steroid-refractory ICIP.

The purpose of this retrospective, single-center descriptive study is to describe the real-world incidence of ICIP and management strategies in oncology patients with a past medical history of pulmonary disease.

## Methods

This single-institution retrospective chart review was conducted within the Houston Methodist Hospital system on patients with cancer that received nivolumab, ipilimumab, pembrolizumab, cemiplimab, or atezolizumab for an FDA approved indication from January 1^st^, 2017 to August 31^st^, 2022. Electronic medical records along with other institution sources (databases, pathology reports, and admission logs) were reviewed to identify potential participants. Patients were excluded if: a patient’s care was transferred to another institution, patient received durvalumab with radiation for early-stage NSCLC, had clinical suspicion of pneumonitis within three months of receiving thoracic radiation, or had previously received an EGFR tyrosine kinase inhibitor (TKI). Patients included in this study met the following inclusion criteria: adult patients 18 years or older diagnosed with any malignancy, received at least two cycles of an immune checkpoint inhibitor, and had a history of an obstructive pulmonary disease prior to introduction of an immune checkpoint inhibitor. A patient’s history of obstructive pulmonary disease was identified using ICD-10 codes and verified through chart review that focused on physician documentation of the disease state, pulmonary function tests (PFTs), medication records, and CT scan records, if available. The primary objective was to evaluate the incidence and management of immune checkpoint inhibitor-induced pneumonitis. Additional data points characterized the pneumonitis events observed and what management strategies were employed to achieve resolution of symptoms. For statistical analysis, descriptive statistics including median, interquartile range, and percentage were used to analyze the baseline characteristics along with the primary and secondary endpoints. Patients were determined to have a diagnosis of immune-induced pneumonitis based on a combination of the following factors: ICIP was indicated on imaging, physicians’ notes indicated diagnosis of ICIP, and management approach indicated a suspicion for ICIP.

## Results

**Figure 1:**
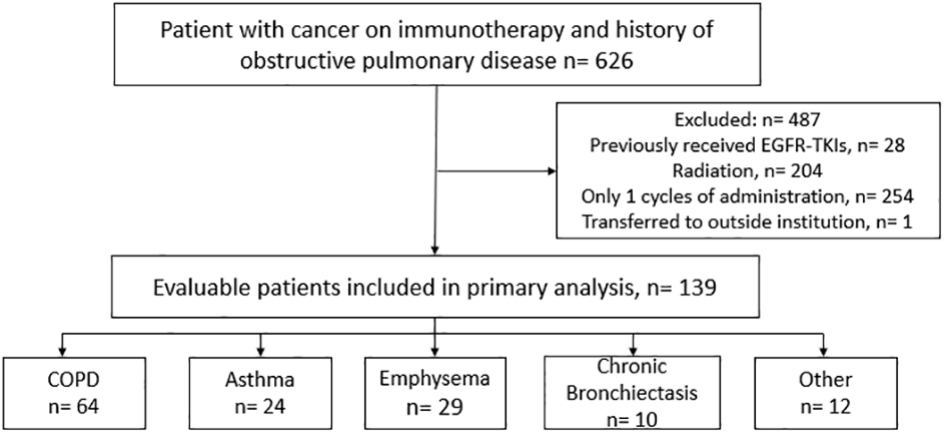
Consort flow diagram.

A total of 626 cancer patients were identified to have a history of an obstructive pulmonary disease prior to introduction of an ICI based on ICD-10 codes and initial ICI administration dates. After applying the exclusion criteria, we reviewed the charts of 139 evaluable patients to identify cases of immune-induced pneumonitis (**Figure 1**). The baseline characteristics for both the overall population and the population that experienced an ICIP event are summarized in **Table 1**. In the overall population, these patients received one of the following immunotherapy agents: pembrolizumab (n=80), nivolumab (n=40), atezolizumab (n=18) or cemiplimab (n=1). The median age, depending on the immunotherapy agent received, is as follows: nivolumab 70 (IQR:13), pembrolizumab 70 (IQR:14), atezolizumab 75 (IQR:12), and cemiplimab 55 (IQR:0), with approximately 50 to 60% of the population being male. The majority (46%) of the population had a history of COPD identified as their underlying obstructive pulmonary disease, followed by asthma and emphysema. The most common oncologic diagnosis in this population was non-small cell lung cancer followed by bladder cancer. Ten patients (7.19%) out of the 139 patients reviewed experienced an ICIP event. Patients who experienced an ICIP event had received either pembrolizumab (n=6; 60%) or nivolumab (n=4; 40%). All of the patients with ICIP had a primary pulmonary lesion (NSCLC, n=8; SCLC, n=1; Mesothelioma, n=1) with the majority having received immunotherapy in combination with chemotherapy (n=6; 60%).

**Table 1:** Baseline demographics.

Baseline Demographics for Evaluable Patients				
Metric	Pembrolizumab	Nivolumab	Atezolizumab	Cemiplimab
**Immunotherapy – no. (%)**	80 (57)	40 (29)	18 (12.9)	1 (0.7)
**Age (yr) – median (IQR)**	70 (14)	70 (13)	75 (12)	55 (0)
**Male Sex – no. (%)**	37 (46.3)	22 (53.6)	12 (66.7)	1 (100)
**Smoking status – no. (%)** Current smoker Former smoker Never smoked	11 (13.7) 56 (70) 13 (16.3)	3 (7.5) 31 (77.5) 6 (15)	3 (16.7) 11 (61.1) 4 (22.2)	0 (0) 1 (100) 0 (0)
**Cancer Staging – no. (%)** Stage I Stage II Stage III Stage IV	10 (12.7) 2 (2.5) 21 (26.6) 47 (58.2)	5 (12.2) 5 (12.2) 11 (26.8) 20 (48.8)	0 (0) 1 (5.6) 6 (3.3) 11 (61.1)	0 (0) 0 (0) 0 (0) 1 (100)
**History of pulmonary disease – no. (%)** COPD Asthma Emphysema Chronic Bronchitis Chronic Bronchiectasis Chronic Bronchiolitis Obstructive Sleep Apnea IPD	38 (47.5) 15 (19) 14 (17.7) 4 (5.1) 5 (6.3) 1 (1.3) 3 (3.8) 0 (0)	20 (48.8) 8 (19.5) 8 (19.5) 1 (2.4) 3 (7.3) 0 (0) 0 (0) 1 (2.4)	6 (33.3) 1 (5.6) 7 (38.9) 1 (5.6) 2 (11.1) 1 (5.6) 0 (0) 0 (0)	1 (100) 0 (0) 0 (0) 0 (0) 0 (0) 0 (0) 0 (0) 0 (0)
**Median Number of Cycles**	5	9	10	9
**Cancer Type – no. (%)** Non-small cell lung cancer Hepatocellular carcinoma Bladder cancer Renal cell carcinoma Small cell lung cancer Other	43 (53.8) 1 (1.3) 9 (11.4) 1 (1.3) 1 (1.3) 25 (31.6)	17 (42.5) 1 (2.4) 4 (9.8) 6 (14.6) 3 (7.3) 9 (21.9)	7 (38.9) 6 (33.3) 1 (5.6) 1 (5.6) 2 (11.1) 1 (5.6)	0 (0) 0 (0) 0 (0) 0 (0) 0 (0) 1 (100)
Baseline Demographics of Patients with Pneumonitis				
Metric	Pembrolizumab		Nivolumab	
**Immunotherapy – no. (%)**	6 (60)		4 (40)	
**Cancer Type – no. (%)** Non-small cell lung cancer Small cell lung cancer Mesothelioma	6 (100) 0 (0) 0 (0)		2 (50) 1 (25) 1 (25)	
**Cancer Staging – no. (%)** Stage III Stage IV	0 (0) 6 (100)		1 (25) 3 (75)	
**Smoking status** Current smoker Former smoker Never smoked	0 (0) 5 (83.3) 1 (16.7)		0 (0) 4 (100) 0 (0)	
**History of pulmonary disease – no. (%)** COPD Asthma Emphysema Chronic Bronchiectasis	2 (33.3) 1 (16.7) 2 (33.3) 1 (16.7)		3 (75) 1 (25) 0 0	
**Combination therapy – no. (%)** Yes No	4 (66.7) 2 (33.3)		2 (50) 2 (50)	

**Table 2** describes the initial immune-induced pneumonitis events that occurred in this population. The severity of the events was graded based on the common terminology criteria for adverse events with the majority meeting criteria for grade 2 pneumonitis (n= 6, 60%). Signs and symptoms of pneumonitis were discovered after a median of 6 cycles in the NSCLC group, 1 cycle in the SCLC group, and 4 cycles in the mesothelioma group. At the time of ICIP identification, 90% of patients had immunotherapy held and were started on either oral or intravenous steroids. Upon initiation of treatment, 60% of patients experienced complete improvement of symptoms and 30% had a partial improvement. One patient showed no improvement in symptoms and was determined to have grade 4 pneumonitis on initial presentation that ultimately proved to be steroid refractory. Once proven to be steroid refractory, this patient received cyclophosphamide and infliximab with no response. Ultimately, the patient succumbed to pneumonitis raising the grade to 5. One additional patient (10%) experienced disease progression while immunotherapy was held for management of ICIP.

**Table 2:** Characterization of patients who developed pneumonitis.

Metric		NSCLC (n=8)	SCLC (n=1)	Mesothelioma (n=1)
**Pneumonitis Grade – no. (%)**	Grade 1 Grade 2 Grade 3 Grade 4 Grade 5	1 (12.5) 4 (50) 2 (25) 0 (0) 1 (12.5)	0 (0) 1 (100) 0 (0) 0 (0) 0 (0)	0 (0) 1 (100) 0 (0) 0 (0) 0 (0)
**Pre-pneumonitis Immunotherapy Cycles – Median (IQR)**		6 (5.25)	1 (0)	4 (0)
**Pneumonitis Recurrence – no. (%)**		3 (37.5)	0 (0)	0 (0)
**Pneumonitis Management – no. (%)**	Immunotherapy held Oral steroids Intravenous steroids Cyclophosphamide Infliximab	7 (87.5) 4 (50) 6 (75) 1 (12.5) 1 (12.5)	1 (100) 0 (0) 0 (0) 0 (0) 0 (0)	1 (100) 0 (0) 1 (100) 0 (0) 0 (0)
**Immunotherapy restarted – no. (%)**	Yes No	5 (62.5) 3 (37.5)	1 (100) 0 (0)	0 (0) 1 (100)
**Post-pneumonitis Immunotherapy Cycles – Median (IQR)**		3 (20)	46 (0)	0 (0)

Of the 10 patients who experienced an ICIP event, 6 (60%) were re-started on immunotherapy as described in **Table 3**. Unfortunately, 3 (50%) of the patients re-started on immunotherapy had a subsequent recurrence of ICIP with the breakdown as follows: nivolumab (n= 2, 33%) and pembrolizumab (n= 1, 17%). All 3 of these patients received higher doses of steroids compared to their initial pneumonitis treatment and 2 (67%) patients required the introduction of intravenous steroids. The median number of cycles in between ICIP events varied widely among the nivolumab and pembrolizumab groups. The nivolumab group displayed a longer median number of cycles at 22 cycles compared to the pembrolizumab group which displayed an almost immediate recurrence after just 1 cycle.

**Table 3:** Characterization of patients who developed recurrence of pneumonitis.

Metric		Nivolumab (n=2)	Pembrolizumab (n=1)
**Combination therapy – no. (%)**	Yes No	1 (50) 1 (50)	1 (100) 0 (0)
**Pre-pneumonitis Immunotherapy Cycles – Median**		2.5	4
**Pneumonitis Initial Management – no. (%)**	Immunotherapy held Oral steroids	2 (100) 2 (100)	1 (100) 0 (0)
**Pneumonitis Recurrence Management – no. (%)**	Immunotherapy held Oral steroids Intravenous steroids	1 (50) 2 (100) 1 (50)	0 (0) 1 (100) 1 (100)
**Immunotherapy restarted – no. (%)**	Yes No	2 (100) 0 (0)	1 (100) 0 (0)
**Immunotherapy Cycles Post-Initial Pneumonitis Event – Median**		22	1

## Discussion

Since the introduction of immunotherapy agents, randomized controlled trials have reported the incidence of immune-induced pneumonitis at about 2 to 2.5%. With little real-world data to support pneumonitis incidence rates, Tiu et al. explored the impact of real-world variability on the incidence of ICIP in the lung cancer population (7). This retrospective cohort study explored the impact of PD-1 and PD-L1 inhibitors on the incidence of pneumonitis and pneumonia as a composite endpoint. The results of this study showed only a marginal increase of 2.49% in risk of pneumonitis and pneumonia-like conditions in the ICI treated versus untreated group (7). However, only about 50% of the ICI treated patients had a prior history of asthma, COPD, or pleural disease at baseline (7). The relatively low representation of patients with a history of pulmonary disease coupled with the use of a composite endpoint makes it difficult to discern the impact pneumonitis events had on the ICI treated population with a prior pulmonary disease history. In our cohort from our hospital system, the incidence of pneumonitis was shown to be higher at 7.19% compared to previous reports from randomized controlled trials and the real-world data reported in the Tiu et al. study. This could be due to our focus on a potentially more vulnerable patient population with an obstructive pulmonary disease history and indicates a potential need to monitor these patients more closely. The higher incidence rate in our cohort is supported by results from the KEYNOTE-001 subgroup analysis which found that pneumonitis occurred more frequently in patients with a history of asthma and chronic obstructive pulmonary disease (COPD) compared to those without (5.4 vs. 3.1%) (3). Our data not only suggests a higher real-world incidence of ICIP events, but it also indicates that any form of obstructive pulmonary disease could potentially be a risk factor for increased risk of ICIP.

Identifying risk factors for the development of pneumonitis has been an ongoing process since the widespread use of immunotherapy began with factors such as recent radiation, use of EGFR TKIs, and combination durvalumab plus radiation clouding the picture. To determine the role of COPD and asthma on the development of ICIP, several studies have investigated these risk factors with mixed results. Chao et al. retrospectively assessed NSCLC patients receiving ICIs to identify risk factors for the development of ICIP and found that COPD was independently associated with a higher ICIP incidence with an odds ratio of 7.194 (CI= 1.130 to 45.798; P-value= 0.037) (8). However, the Zeng et al. study, that collected data from a very similar population to the Chao et al. study, did not see a link between co-existing COPD and a higher risk of ICIP but did support a higher incidence of pneumonitis in their subgroup of patients with a history of pulmonary fibrosis (8, 9). The lack of support for the hypothesis that history of COPD contributes to higher incidences of ICIP development may be explained by the smaller population size evaluated in that trial. Nonetheless, our finding within our population who possessed a variety of pulmonary disorders corroborates this trial’s implication of pulmonary disease as a potential contributor to ICIP occurrence and establishes a history of obstructive pulmonary disease as a risk factor for pneumonitis that clinicians should be wary of prior to initiating ICI. The top 3 pulmonary diseases represented in our study were COPD (n= 64, 46%), emphysema (n= 29, 21%), and asthma (n= 24, 17%). In addition to our findings, Galant et al. studied 187 patients, 26 of which had an asthma diagnosis (10). Out of those 26 patients, 3 patients developed ICIP which accounted for 11.5% of the asthma patients in the population (10). All 3 asthma patients presented with severe pneumonitis that were classified as grade 3 and 4 reactions (10). The severity of the presentation coupled with the high percentage of asthma patients found to have developed pneumonitis implicates asthma as a potential serious risk factor to consider in the setting ICIP. Our data supports both COPD and asthma as risk factors for ICIP due to our high percentage of both pulmonary diseases in our base population. In addition, our findings also highlight that patients with an underlying diagnosis of emphysema and chronic bronchiectasis are also at risk for ICIP. These findings necessitate closer monitoring and follow-up after initiation of ICI treatment in patients with an underlying obstructive pulmonary disease.

Tiu et al. investigated the real-world impact of pneumonitis in lung cancer in which glucocorticoids were frequently administered to their patients with suspected ICIP. 212 (83.5%) of their 254 patients that experienced an ICIP event received oral prednisone within 30 days following diagnosis (7). Intravenous methylprednisolone was administered to 145 patients (57.1%) with high-grade adverse effects in the same 30-day interval post-diagnosis (7). Of the 225 patients that received an ICI, 158 (70.2%) discontinued treatment within 90 days of pneumonitis diagnosis (7). In the initial management of ICIP for our population, 4 patients (40%) received oral prednisone tapering over twelve to fifteen days and 6 patients (60%) received intravenous methylprednisolone with one patient receiving both oral and intravenous steroids in the management of their first pneumonitis event. Additionally, 9 patients (90%) had immunotherapy held quickly after ICIP diagnosis. Compared to the Tiu et al. study, our population was more likely to have their immunotherapy agent held and intravenous steroids started which resulted in a complete resolution of symptoms in 6 patients (60%) and partial resolution of symptoms in 3 patients (30%). Based on the data from both studies, there is continued evidence of the effectiveness of steroids for the management of initial ICIP episodes. Although holding immunotherapy and initiating steroids assisted in resolving ICIP symptoms for some patients, Tiu et al. reported a high rate of mortality at 32.7% in their population within 90 days of ICIP diagnosis due to a combination of disease progression and high-grade ICIP (7). At completion of the chart review time frame, 1 patient (10%) had died due to respiratory failure attributed to ICIP after treatment with steroids, cyclophosphamide, and infliximab.

In our study, 6 patients were re-started on immunotherapy after resolution of their initial ICIP symptoms. However, 3 patients (50%) experienced a recurrent episode of ICIP upon re-initiation requiring longer, higher doses of steroids along with more frequent use of intravenous steroids. The timing of these recurrent episodes varied from a median of 1 month to a little over one-year post-ICI therapy re-introduction. The wide time frame for recurrence can be attributed to the small number of patients in our population that experience a recurrence. Two of the 3 patients who experienced a recurrence received only 1 to 3 cycles of immunotherapy while one patient received 41 cycles prior to a subsequent ICIP event. In a study conducted by Tao et al., the authors reported a median ICIP recurrence onset of 2.78 months which most closely aligns with the timeframe in which 2 of our patients presented with recurrence (11). This indicates that the majority of recurrence cases have the tendency to occur within a 2-to-3-month time interval of treatment re-initiation. Another study by Dolladille et al. looked at a total of 6,123 cases of immune-induced adverse effects (irAEs) and found that 452 (7.4%) of irAEs were associated with ICI rechallenges with 28.8% of irAE recurrences involving the same organ as the initial irAE identified (12). Additionally, pneumonitis was specifically associated with a higher recurrence rate compared with other irAEs (12). This further supports our findings of a higher pneumonitis recurrence rate among the patients in our population. Additionally, two patients (20%) experienced disease progression while immunotherapy was held for management of ICIP. Unfortunately, due to the evidence that supports high levels of recurrence and risk for disease progression while holding immunotherapy further investigations are needed into how to safely re-introduce ICI agents in this patient population.

Our study was limited by the small population size and therefore the small number of ICIP events reported which prohibits us from drawing statistically significant conclusions from the data presented. However, the data collected in this study does show trends toward pre-existing obstructive pulmonary disease having an impact on ICIP events rates, presentation, and management which necessitates a need for future prospective large-scale studies to further our knowledge of ICIP management. Although steroids and holding immunotherapy have been proven strategies for resolving ICIP for most patients, further research needs to elucidate management strategies that would be effective in the setting of rechallenge, steroid refractory ICIP, and ultimately reduce the risk of recurrence associated with holding ICI therapy and utilizing immunosuppressive doses of steroids. Our identification of ICIP cases was also dependent on manual chart review due to the retrospective nature of this study, which relied heavily on physician diagnosis documentation, medication administration records and imaging interpretation which may not be as reliable as data collected in a prospective manner. Future studies could evaluate the effects of ICIP on survival outcomes and the contribution of all obstructive pulmonary disease on incidence of ICIP.

## Conclusion

Based on our findings, the real-world incidence of ICIP in patients with an underlying obstructive pulmonary disease history is higher than previously described in literature. Due to this increased concern for the development of ICIP in this population, more frequent monitoring and follow-up may be warranted to catch the development of ICIP at an earlier grade. Furthermore, a history of an obstructive pulmonary disorder should be a part of the risk versus benefit discussion surrounding use of an immunotherapy agent prior to patient initiation. Lastly, the high rates of ICIP recurrence reported coupled with the lack of effective management options for steroid refractory ICIP should be considered prior to re-introduction of ICIP therapy.

## Data availability statement

The raw data supporting the conclusions of this article will be made available by the authors, without undue reservation.

## Ethics statement

The studies involving humans were approved by Houston Methodist Research Institute IRB. The studies were conducted in accordance with the local legislation and institutional requirements. Written informed consent for participation was not required from the participants or the participants’ legal guardians/next of kin in accordance with the national legislation and institutional requirements.

## Author contributions

EA: Conceptualization, Data curation, Formal Analysis, Writing – original draft. GU: Conceptualization, Supervision, Writing – review & editing. VA: Supervision, Writing – review & editing. EB: Supervision, Writing – review & editing.
